# Factors for achieving target serum uric acid levels after initiating urate-lowering therapy in patients with gout: results from the ULTRA registry

**DOI:** 10.1038/s41598-023-47790-6

**Published:** 2023-11-22

**Authors:** Hyunsue Do, Hyo Jin Choi, Byoongyong Choi, Chang-Nam Son, Sang-Hyon Kim, Se Rim Choi, Ji Hyoun Kim, Min Jung Kim, Kichul Shin, Hyun-Ok Kim, Ran Song, Sung Won Lee, Joong Kyong Ahn, Seung-Geun Lee, Chang Hoon Lee, Kyeong Min Son, Ki Won Moon

**Affiliations:** 1 Division of Rheumatology, Department of Internal Medicine, Kangwon National University School of Medicine, 1 Kangwondaehak-gil, Chuncheon, 24341 South Korea; 2 Division of Rheumatology, Department of Internal Medicine, Gil Medical Center, Gachon University College of Medicine, Incheon, South Korea; 3 Division of Rheumatology, Department of Internal Medicine, Seoul Metropolitan Seoul Medical Center, Seoul, South Korea; 4 Division of Rheumatology, Department of Internal Medicine, Eulji University School of Medicine, Uijeongbu, South Korea; 5 Division of Rheumatology, Department of Internal Medicine, Keimyung University School of Medicine, Daegu, South Korea; 6 Division of Rheumatology, Department of Internal Medicine, Seoul National University Bundang Hospital, Seongnam, South Korea; 7 Division of Rheumatology, Department of Internal Medicine, Chungbuk National University Hospital, Cheongju, South Korea; 8 Division of Rheumatology, Department of Internal Medicine, Seoul Metropolitan Government-Seoul National University Boramae Medical Center, Seoul, South Korea; 9 Division of Rheumatology, Department of Internal Medicine, Gyeongsang National University School of Medicine, Jinju, South Korea; 10 Division of Rheumatology, Department of Internal Medicine, Kyung Hee University, Seoul, South Korea; 11 Division of Rheumatology, Department of Internal Medicine, Soon Chun Hyang University Hospital, Cheonan, South Korea; 12 Division of Rheumatology, Department of Internal Medicine, Kangbuk Samsung Hospital, Sungkyunkwan University School of Medicine, Seoul, South Korea; 13 Division of Rheumatology, Department of Internal Medicine, Pusan National University School of Medicine, Pusan National University Hospital, Busan, South Korea; 14 Division of Rheumatology, Department of Internal Medicine, Wonkwang University School of Medicine, Iksan, South Korea; 15 Division of Rheumatology, Department of Internal Medicine, Hallym University Dongtan Sacred Heart Hospital, Hwaseong-si, Gyeonggi-do South Korea

**Keywords:** Rheumatology, Gout

## Abstract

Achieving target serum uric acid (SUA) levels is important in gout management. Guidelines recommend lowering SUA levels to < 6 mg/dL; however, many patients fail to reach this target, even with uric acid-lowering therapy (ULT). This study investigated clinical characteristics of target SUA achievers among Korean patients with gout. This study used data from the ULTRA registry, a nationwide inception cohort established in September 2021 that enrolls patients with gout who initiate ULT. Demographic, clinical, and laboratory data were collected at baseline; the 6-month follow-up. Patients were divided into two groups: target achievers (SUA level < 6 mg/dL at 6 months) and non-achievers. The mean participant (N = 117) age was 56.1 years, and 88.0% were male. At 6 months, 83 patients (70.9%) reached target SUA levels. Target achievers had better drug adherence (≥ 80%) to ULT (97.6% vs. 76.5%; *p* < 0.01) than non-achievers. Target non-achievers had a higher percentage of a family history of gout (32.4% vs. 10.8%; *p* < 0.01) and less antihypertensive agent use (38.2% vs. 59.0%; *p* = 0.03) than target achievers. Multivariate regression analysis revealed that good adherence to ULT, the absence of a family history of gout, and antihypertensive agent use were key factors associated with achieving target SUA levels at 6 months.

## Introduction

Gout is one of the most common types of inflammatory arthritis and its incidence has been increasing in Korea, especially in the young population^1^. Several guidelines for the management of gout recommend reducing serum uric acid (SUA) levels to < 6 mg/dL^2,3^. Achieving target SUA levels is important to manage gout. Tight control of uric acid levels to below 6 mg/dL has been associated with a reduction in gout attacks, comorbidities^4^, size of tophi^5^, and the prevention of structural damage^6^. However, many patients fail to reach the target despite uric acid-lowering therapy (ULT)^7^. A cross-sectional study from Japan reported that 37.5% of patients with gout achieved target SUA levels^8^, and another study from the Philippines showed that 37.7% and 26.2% of patients reached target SUA levels at 6 and 12 months, respectively^9^. Several clinical factors have been associated with reaching target SUA levels. Singh et al. reported that a lower Charlson comorbidity index and more outpatient visit days were associated with a higher rate of reaching the target SUA level^10^. Another study revealed that the clinical factors associated with achieving target SUA levels in 12 months were employment, baseline SUA level, and age at gout onset^9^. However, most of these studies were retrospective, and there have been no conclusive findings on this topic. This study aimed to investigate the characteristics of target SUA achievers and the key factors for achieving target SUA levels in Korean patients with gout using prospective cohort data.

## Results

A total of 117 patients were enrolled in this study. The mean (± SD) age was 56.1 (± 18.2) years, and 88.0% were male. At 6 months, 83 patients (70.9%) reached the target SUA level. Table 1 shows the baseline clinical and laboratory characteristics of the patients with gout who did and did not achieve target SUA levels at 6 months. The mean (± SD) SUA level of the achiever group was 4.7 (± 0.8) mg/dL, and that of the non-achiever group was 7.7 (± 1.7) mg/dL. The achiever group had a lower proportion of a familial history of gout (10.8% vs. 32.4%; *p* < 0.01) and better adherence to ULT (97.6% vs. 76.5%; *p* < 0.01). Regarding ULT agents, lower allopurinol use, slightly higher febuxostat use, and higher benzbromarone use were observed in the achiever group than in the non-achiever group (allopurinol, 14.5% vs. 32.4%; febuxostat, 69.5% vs. 64.7%; benzbromarone, 15.7% vs. 2.9%; *p* < 0.01). We permitted the combination use of ULT agents, but there were no patients who used two or more ULT. The achiever group used antihypertensive agents more frequently (59.0% vs. 38.2%; *p* = 0.03) than the non-achiever group did. Among the antihypertensive agents, ARB and CCB users were more common in the achiever group than in the non-achiever group, although the differences were not significant (ARB: 61.2% vs. 46.2%; *p* = 0.08; CCB: 49.0% vs. 30.8%; *p* = 0.06). The BMI, smoking status, and alcohol consumption did not differ significantly between the two groups. Laboratory results, except for the SUA level and patient-reported outcomes regarding quality of life (GIS and EQ-5D) were also not significantly different (Supplemental Table S1). Univariate regression analysis showed family history of gout, adherence to ULT, and antihypertensive agent use were statistically significant factors (Supplemental Table S2). Multivariate regression analysis revealed that the absence of a family history of gout, good adherence to ULT, and antihypertensive agent use were significant factors associated with achieving the target SUA level at 6 months (Table 2).

**Table 1:** Clinical and laboratory characteristics of target achievers and non-achievers at baseline and 6 months (n = 117).

Characteristics	Total (N = 117)	Achievers (N = 83)	Non-achievers (N = 34)	*p* value
Age (years), mean ± SD	56.1 ± 18.2	57.2 ± 17.5	53.35 ± 19.8	0.30
Male (%)	103 (88.0)	72 (86.7)	31 (91.2)	0.37
BMI (kg/m^2^), mean ± SD	26.3 ± 3.6	26.4 ± 3.7	26.2 ± 3.6	0.72
Systolic BP, mean ± SD	131.3 ± 17.4	132.4 ± 16.5	128.5 ± 19.5	0.27
Diastolic BP, mean ± SD	80.2 ± 13.2	80.4 ± 12.7	79.7 ± 14.6	0.79
Disease duration (years), mean ± SD	4.5 ± 6.3	4.52 ± 6.0	4.47 ± 6.9	0.97
Gout flares ≥ 2/year (%)	22 (18.8)	14 (16.9)	8 (23.5)	0.27
Presence of tophi (%)	26 (22.2)	18 (21.7)	8 (23.5)	0.50
Erosion on joint X ray (%)	25 (21.4)	21 (25.3)	4 (11.8)	0.09
Familial history of gout (%)	20 (17.1)	9 (10.8)	11 (32.4)	< 0.01**
History of urinary stones (%)	7 (6.0)	4 (4.8)	3 (8.8)	0.41
Smoking				
Non-smoker (%)	45 (38.5)	34 (41.0)	11 (32.4)	0.39
Past smoker (%)	45 (38.5)	31 (37.3)	14 (41.2)	
Current smoker (%)	27 (23.1)	18 (21.7)	9 (26.5)	
Alcohol				
Non-drinker (%)	27 (23.1)	19 (22.9)	8 (23.5)	0.41
Past drinker (%)	24 (20.5)	14 (16.9)	10 (29.4)	
Current drinker (%)	66 (56.4)	50 (60.2)	16 (47.1)	
Acute flare within 7 days (%)	59 (50.4)	42 (50.6)	17 (50.0)	0.91
Acute flare joint count, mean ± SD	1.36 ± 0.9	1.4 ± 1.0	1.3 ± 0.5	0.72
Previous flare number, mean ± SD	4.2 ± 5.1	4.0 ± 3.8	4.8 ± 7.5	0.59
Fulfillment of 2015 gout classification criteria (%)	117 (100.0)	83 (100.0)	34 (100.0)	1.00
MSU positive (%)	11 (9.4)	5 (6.0)	6 (17.6)	0.19
ULT agents (%)				
Allopurinol	23 (19.7)	12 (14.5)	11 (32.4)	< 0.01**
Febuxostat	80 (68.4)	58 (69.6)	22 (64.7)	
Benzbromarone	14 (12.0)	13 (15.7)	1 (2.9)	
Combined medications, n (%)				
Diuretics	17 (14.5)	12 (14.5)	5 (14.7)	0.59
Antihypertensive agents	62 (53.0)	49 (59.0)	13 (38.2)	0.03*
ACEi	4 (6.5)	3 (6.1)	1 (7.7)	0.70
ARB	36 (58.1)	30 (61.2)	6 (46.2)	0.08
CCB	28 (45.2)	24 (49.0)	4 (30.8)	0.06
β-blocker	9 (14.5)	6 (12.2)	3 (23.1)	0.72
α-blocker	1 (1.6)	1 (2.0)	0 (0.0)	1.00
Aspirin	14 (12.0)	10(12.0)	4 (11.8)	0.62
Antiplatelet agent	9 (7.7)	4 (4.8)	5 (14.7)	0.08
Anticoagulant	11 (9.4)	9 (10.8)	2 (5.9)	0.33
Hypoglycemic agent	19 (16.2)	12 (14.5)	7 (20.6)	0.29
Statin	30 (25.6)	24 (28.9)	6 (17.6)	0.15
Laboratory results, mean ± SD				
Initial serum uric acid (mg/dL)	8.8 ± 1.8	8.7 ± 1.6	9.1 ± 2.3	0.33
Characteristic at 6 months				
Adherence to ULT for 6 months				
≥ 80%	107 (91.5)	81 (97.6)	26 (76.5)	< 0.01**
< 80%	10 (8.5)	2 (2.4)	8 (23.5)	
Laboratory results, mean ± SD				
Serum uric acid at 6 months (mg/dL)	5.6 ± 1.8	4.7 ± 0.8	7.7 ± 1.7	< 0.01**

*BMI* body mass index, *BP* blood pressure, *MSU* monosodium urate, *ULT* urate-lowering therapy, *XOI* xanthine oxidase inhibitor, *ACEi* angiotensin-converting enzyme inhibitor, *ARB* angiotensin receptor blocker, *CCB* calcium channel blocker, *SD* standard deviation.

*p < 0.05, **p < 0.01.

**Table 2:** Factors associated with achieving target serum uric acid levels at 6 months.

	Univariate			Multivariate		
	OR	95% CI	*p* value	OR	95% CI	*p* value
Age	1.01	0.99–1.03	0.30	1.00	0.97–1.03	0.95
Sex						
Female	Ref			Ref		
Male	0.63	0.17–2.43	0.51	0.77	0.14–4.23	0.77
Alcohol						
Non-drinker	Ref			Ref		
Past drinker	0.59	0.19–1.88	0.37	0.38	0.87–1.68	0.20
Current drinker	1.32	0.48–3.58	0.59	0.93	0.24–3.56	0.91
Family history of gout	0.26	0.10–0.70	< 0.01**	0.19	0..6–0.67	< 0.01**
Use of antihypertensive agents	2.33	1.03–5.28	0.04*	3.44	1.09–10.80	0.04*
Adherence to ULT ≥ 80%	12.31	2.46–61.67	< 0.01**	6.81	1.20–38.58	0.03*
ULT						
Allopurinol	Ref			Ref		
Febuxostat	2.42	0.93–6.27	0.07	1.77	0.57–5.48	0.33
Benzbromarone	10.92	1.33–106.73	0.03*	9.12	0.71–93.79	0.09

*ULT* urate-lowering therapy, *OR* odds ratio, *CI* confidence interval.

*p < 0.05, **p < 0.01.

## Discussion

Achieving and maintaining the target SUA level is important for the proper management of patients with gout. One Korean study followed 200 patients for an average of 7.6 years and reported that the incidence of new chronic diseases, including hypertension, diabetes, cardiovascular disease, and urolithiasis, was lower in the achiever group, who also experienced a reduction in the yearly rate of gout flares^4^. An observational cohort study involving 26,341 gout patients in the United States showed that achieving the target SUA level was associated with a reduced risk of renal function deterioration^11^. However, if the target SUA level is not reached, the clinical course of gout may deteriorate. One prospective study, in which 1193 European patients were followed-up for 2 years, reported that patients who did not reach the target SUA level of < 6 mg/dL had an increased mortality rate, especially cardiovascular mortality^12^. Therefore, physicians need to be aware of the clinical factors associated with achieving target SUA levels in patients with gout.

Our study showed that no familial history of gout, using antihypertensive agents, and good drug adherence were clinical factors for achieving the target SUA level at 6 months. Various factors have been associated with achieving target SUA levels. A study analyzing the US Veteran Affairs database reported that an older age, normal BMI, low Charlson index, rheumatologist as the main provider, easy healthcare accessibility, and low baseline SUA were associated with achieving target SUA levels^13^. A study involving New Zealand patients showed that the ULT agent dose, patient confidence, female sex, and ethnicity were independent factors for achieving target SUA levels^14^. Another study using Japanese health insurance claims data showed that old age, female sex, higher ULT agent dose, good drug adherence, more comorbidities, and the use of antidiabetic drugs were significant factors for achieving target SUA levels^15^. In addition, renal impairment and diuretic use reduced the achievement of target SUA levels. The significant factors presented in each study differed, likely because the characteristics of the enrolled patients differed.

In our study, approximately 70% of patients reached the target SUA level at 6 months. This percentage was higher than that of other studies because the cohort composition in our study was different than that in other studies. The participating hospitals in our study were mainly secondary and tertiary medical institutions, and the patient characteristics differed from those in primary hospitals. The adherence to ULT at 6 months for all patients was > 90%, which is higher than that reported in other studies. The fact that all investigators who participated in this study were rheumatologists might have contributed to the increased adherence rates. When the primary healthcare provider is a rheumatologist, the rate of achieving the target SUA level is higher than that with primary care physicians^16^. Another reason for the high adherence rate was that the follow-up period was relatively short (6 months). If the patient is observed for a longer period, adherence will decrease.

Our study showed that a familial history of gout was associated with a reduced achievement of the target SUA level. If patients with gout have a family history, disease onset generally occurs at a young age, and the risk of progression to chronic tophaceous gout also increases^17^. Moreover, they have a greater chance of having polymorphisms in genes encoding urate transporters such as ABCG2. These genetic polymorphisms are associated with a poor response to ULT^18^. Therefore, physicians need to pay more attention to monitoring SUA levels in patients with a familial history of gout. Adherence to ULT is also an important issue in achieving target SUA levels. A study involving US insurance data showed that good adherence to ULT and lower baseline SUA levels were strong predictors of achieving target SUA levels^19^. Another Malaysian study reported that ULT nonadherence and the presence of tophi were independent factors for the failure to achieve target SUA levels^20^. Various methods have been proposed to improve ULT adherence in patients with gout, including daily text messaging and patient-focused and nurse- and rheumatologist-led education^21^. Most interventions have focused on enhancing the communication between patients and healthcare providers. Using the various intervention methods available at each medical facility may improve the ULT adherence.

The pattern of ULT use was different in both groups in our study; however, it was not a significant factor in the multivariate logistic regression analysis. Generally, all ULT agents are effective in achieving the target SUA level, although febuxostat is known to be more effective and faster-acting than allopurinol^22^. Benzbromarone is not prescribed as often as allopurionol or febuxostat but is effective in reducing SUA levels. A prospective randomized study comparing the efficacy of febuxostat and benzbromarone showed that the proportion of patients who achieved target SUA levels was not significantly different (febuxostat 39.5% vs. benzbromarone 35.7%)^23^. Our dataset was probably not large enough to address this issue, but we will analyze it later, as more data are accumulated.

Interestingly, the use of antihypertensive drugs was a significant factor affecting target SUA levels. Among antihypertensive agents, ARB and CCB were more commonly used in the achiever group. ARB, especially losartan, and CCB are the recommended agents for gout patients with hypertension, according to the guidelines^2^. Each antihypertensive drug has a different effect on SUA levels. One study reported that diuretics, β-blockers, and α-blockers increased SUA levels, but CCB, ACEi, and ARB did not increase SUA levels^24^. ARBs, especially losartan, can help patients achieve target SUA levels because of their uricosuric effect^25^, and dihydropyridine CCBs reduce SUA levels^26^. A large epidemiological study reported that losartan and CCB are associated with a reduced risk of incident gout^27^. If patients with gout have hypertension, ARB, especially losartan and CCB, should be recommended first.

The strength of this study is that we analyzed data from a prospective cohort. Prospective cohort studies have many advantages over retrospective studies, including the ability to reduce biases and collect more accurate data. In addition, we collected data from 15 centers across the country. Because each center represents a region, the subjects in our study might represent the entire nation. However, this study had some limitations. First, we enrolled patients from secondary or tertiary hospitals, and selection bias could have occurred. Second, these results may not reflect the real world, as we could not collect data from patients who did not visit the hospital after 6 months. The third limitation is that the number of enrolled patients was small, and the follow-up period was relatively short. Therefore, we could not find long-term outcome differences according to achieving the target SUA level. However, our cohort study is ongoing, and we expect that these issues will be resolved in the future. Fourth, drug adherence was measured after 6 months, whereas other factors were measured at baseline because we could not measure drug adherence at the time of starting ULT. We chose drug adherence as an independent variable in the regression analysis because it is a well-known factor in achieving target SUA levels. Yet, the results should be interpreted with caution. Our study showed that drug adherence is significantly associated with achieving target SUA levels, not a direct causal relationship.

In summary, we identified key factors associated with target SUA levels, including no familial history of gout, good adherence to ULT, and use of antihypertensive agents. These findings suggest that adherence to ULT is important for achieving the target SUA level and that more attention should be paid to patients with a familial history of gout. Antihypertensive agents, especially ARB and CCB, can help patients with gout achieve target SUA levels.

## Methods

### Study population

This study was based on data from ‘The Urate-Lowering TheRApy in gout’ (ULTRA) registry, which is supported by the Korean Ministry of Health and Welfare (https://cris.nih.go.kr/, KCT0007395)^28^. The enrollment of participants and data collection from the ULTRA registry are ongoing. This inception cohort was established in September 2021 and consists of Korean patients with gout treated at 15 centers nationwide. The participants are aged 18 years or older and fulfill the 2015 American College of Rheumatology/European League Against Rheumatism (ACR/EULAR) classification criteria for gout. The enrollment criteria include at least one of the following criteria: two or more gout flares in a year, presence of tophi, erosions present in hand and foot X-rays, and other reasons that ULT are necessary, according to the investigator’s opinion. All patients were required to sign informed consent forms and complete questionnaires regarding demographics, medical history, comorbidities, and quality of life. The data were saved and managed in an electronic recording system, the Internet-based Clinical Research and Trial Management System (iCReaT), developed by the Korean Center for Disease Control (https://icreat.nih.go.kr). Visit schedules were at baseline, 6 months, 1 year, and every year thereafter. Patients were assigned to the ULT (administration of allopurinol, febuxostat, or benzbromarone) or observation group according to a shared decision between the patient and investigator. Only baseline and 6 month data of the patients who underwent ULT were included in this study. This study was conducted in compliance with the World Medical Association Declaration of Helsinki and approved by the Institutional Review Board of Kangown National University Hospital. The study is reported according to the STROBE statement.

### Data collection

In this study, we used demographic, clinical, medication, and laboratory data at baseline and 6 months. Baseline demographic data included age, sex, body mass index (BMI), blood pressure, smoking, and alcohol status. Clinical data included disease duration, gout flares more than twice a year, presence of tophi, erosion on joint radiography, familial history of gout (including second-degree relatives), acute flare within 7 days, acute flare joint count, previous gout flare number, fulfillment of the 2015 gout classification criteria, and positivity of monosodium urate crystals on polarized microscopy. Medication data included ULT agent-initiated and combined medications (diuretics, antihypertensive agents, aspirin, antiplatelet agents, anticoagulants, hypoglycemic agents, and statins). For antihypertensive agents, the class of each drug was recorded, including angiotensin-converting inhibitor (ACEi), angiotensin receptor blocker (ARB), calcium channel blocker (CCB), β-blocker, and α-blocker. For combination formula drugs, each drug was regarded as a single agent. Quality of life was measured using the Gout Impact Scale (GIS) and Euro-Quality of Life Five Dimension (EQ-5D) scale. Levels of hemoglobin, white blood cells, platelets, aspartate aminotransferase, alanine aminotransferase, high-density lipoprotein, low-density lipoprotein, triglycerides, blood urea nitrogen, creatinine, estimated glomerular filtration, erythrocyte sedimentation rates, C-reactive protein, and SUA were measured at baseline. The 6-month SUA levels and drug adherence to ULT were obtained from the data collected at 6 month. The drug adherence to ULT was measured using the medication possession ratio (MPR). An MPR ≥ 0.8 was considered good drug adherence. Patients were divided into two groups according to SUA levels at 6 months: those who reached SUA levels < 6 mg/dL (achievers) and those who did not (non-achievers).

### Statistical analysis

We compared the clinical and laboratory characteristics of both groups and analyzed the clinical factors associated with achieving target SUA levels at 6 months. Continuous variables are displayed as mean ± standard deviation (SD) and were analyzed by the Student’s t-test. Categorical variables are described as numbers and percentages and were compared using the chi-squared test. Logistic univariate regression analysis was performed between the two groups. Statistically significant variables were selected by univariate analysis were included in the multivariate regression analysis. In addition to statistically significant variables, well-known essential variable including age, sex, and alcohol consumption were also included in the multivariate analysis, Clinical factors associated with achieving the target SUA level were identified using odds ratios (OR) and 95% confidence intervals (CI) for each item using univariate and multivariate models. *p* values < 0.05 were considered statistically significant. All statistical analyses were performed using Statistical Package for Social Sciences, version 26 (SPSS, Chicago, IL, USA).

### Ethics declarations

The present study protocol was reviewed and approved by the Institutional Review Board of Kangown National University Hospital (approval No. KNUH-2023–03-010-001). Informed consent was submitted by all subjects when they were enrolled.

### Supplementary Information

Supplementary Tables.

## Data Availability

Data are available on reasonable request to the corresponding author.
