# Supplementary material for: Factors for achieving target serum uric acid levels after initiating urate-lowering therapy in patients with gout: results from the ULTRA registry
Source: Sci Rep. 2023 Nov 22;13:20511. doi: 10.1038/s41598-023-47790-6 (PMC10665459; doi:10.1038/s41598-023-47790-6)
Supplement: Supplementary file 1 — Supplementary Tables. [file 41598_2023_47790_MOESM1_ESM.docx]

Table S1. Baseline laboratory and patient-reported outcomes of target achievers and non-achievers (N=117)

| Characteristics | Total (N=117) | Achievers (N=83) | Non-achievers (N=34) | *P* value |
| --- | --- | --- | --- | --- |
| Laboratory results, mean ± SD |  |  |  |  |
| HB (g/dL) | 13.6 ± 2.2 | 13.4 ± 2.2 | 13.8 ± 2.3 | 0.47 |
| WBC (/uL) | 7.8 ± 2.4 | 8.1 ± 2.5 | 7.2 ± 2.0 | 0.11 |
| Platelet (10^3^/uL) | 259.2 ± 85.8 | 268.8 ± 87.6 | 239.5 ± 79.7 | 0.12 |
| AST (U/L) | 30.7 ± 16.3 | 29.6 ± 14.6 | 33.3 ± 19.9 | 0.28 |
| ALT (U/L) | 27.8 ± 29.5 | 34.6 ± 21.2 | 45.2 ± 43.1 | 0.19 |
| Total bilirubin (mg/dL) | 0.7 ± 0.3 | 0.6 ± 0.3 | 0.7 ± 0.4 | 0.26 |
| Total cholesterol (mg/dL) | 184.8 ± 49.0 | 184.5 ± 48.1 | 185.5 ± 51.8 | 0.93 |
| HDL (mg/dL) | 44.8 ± 10.9 | 44.4 ± 10.9 | 46.2 ± 11.3 | 0.56 |
| LDL (mg/dL) | 110.8 ± 44.0 | 109.2 ± 42.3 | 116.0 ± 50.2 | 0.58 |
| TG (mg/dL) | 228.0 ± 239.1 | 242.3 ± 267.4 | 184.4 ± 111.5 | 0.38 |
| BUN (mg/dL) | 19.6 ± 10.5 | 20.0 ± 10.2 | 18.6 ± 11.4 | 0.52 |
| Creatinine (mg/dL) | 1.1 ± 0.3 | 1.1 ± 0.3 | 1.2 ± 0.4 | 0.24 |
| EGFR (mL/min/1.73㎡) | 77.0 ± 26.0 | 76.8 ± 25.7 | 77.4 ± 27.1 | 0.91 |
| ESR (mm/hr) | 23.0 ± 24.0 | 23.3 ± 24.4 | 22.4 ± 23.6 | 0.88 |
| CRP (mg/dL) | 1.4 ± 3.4 | 1.5 ± 3.8 | 1.4 ± 2.6 | 0.90 |
| Gout impact scale (0-100), mean ± SD | | | | |
| Gout concern overall | 78.0 ± 20.0 | 78.4 ±21.6 | 83.8 ± 15.2 | 0.18 |
| Gout medication side effects | 62.1 ± 23.7 | 61.6 ±24.4 | 63.2 ± 22.2 | 0.74 |
| Unmet gout treatment needs | 47.2 ± 14.1 | 46.8 ± 13.7 | 48.3 ± 15.2 | 0.60 |
| Well-being during attack | 55.7 ± 26.3 | 56.6 ±26.8 | 53.1 ± 25.1 | 0.55 |
| Gout concern during attack | 58.9 ± 22.7 | 59.3 ± 23.5 | 57.9 ± 21.0 | 0.76 |
| EQ-5D-3L (1-3), mean ± SD |  |  |  |  |
| Mobility | 1.4 ± 0.6 | 1.4 ± 0.5 | 1.4 ± 0.6 | 0.90 |
| Self-care | 1.1 ±0.4 | 1.1 ± 0.4 | 1.1 ± 0.4 | 0.68 |
| Usual activity | 1.4 ± 0.5 | 1.4 ± 0.5 | 1.4 ± 0.6 | 0.83 |
| Pain/discomfort | 1.7 ± 0.6 | 1.7 ± 0.6 | 1.7 ± 0.7 | 0.86 |
| Anxiety/depression | 1.3 ± 0.6 | 1.3 ± 0.6 | 1.3 ± 0.5 | 0.72 |

HB, hemoglobin; WBC, white blood cell; AST, aspartate aminotransferase; ALT, alanine aminotransferase; HDL, high density lipoprotein; LDL, low density lipoprotein; TG, Triglyceride; BUN, blood urea nitrogen, EGFR, estimated glomerular filtration rate; ESR, erythrocyte sedimentation rate; CRP, C-reactive protein; EQ-5D-3L, EuroQol-5 dimension-3 level

Table S2. Univariate analysis of factors associated with achieving target serum uric acid levels at 6 months

|  | OR | 95% CI | *P* value |
| --- | --- | --- | --- |
| Age | 1.01 | 0.99-1.03 | 0.30 |
| Sex | | | |
| Female | Ref |  |  |
| Male | 0.63 | 0.17-2.43 | 0.51 |
| BMI | 1.02 | 0.91-1.14 | 0.72 |
| Systolic BP | 1.01 | 0.99-1.04 | 0.27 |
| Diastolic BP | 1.00 | 0.97-1.03 | 0.78 |
| Disease duration | 1.00 | 0.93-1.06 | 0.97 |
| Gout flares ≥ 2/year | 1.53 | 0.57-4.09 | 0.40 |
| Presence of tophi | 0.89 | 0.34-2.32 | 0.82 |
| Erosion on joint X ray | 2.50 | 0.79-7.94 | 0.12 |
| Familial history of gout | 0.26 | 0.10-0.70 | <0.01** |
| History of urinary stones | 0.52 | 0.11-2.47 | 0.41 |
| Smoking | | | |
| Non-smoker | Ref |  |  |
| Past smoker | 0.72 | 0.28-1.81 | 0.48 |
| Current smoker | 0.61 | 0.20-1.81 | 0.37 |
| Alcohol | | | |
| Non-drinker | Ref |  |  |
| Past drinker | 0.59 | 0.19-1.88 | 0.37 |
| Current drinker | 1.32 | 0.48-3.58 | 0.59 |
| Acute flare within 7 days | 1.05 | 0.47-2.34 | 0.91 |
| Acute flare joint count | 1.11 | 0.54-2.30 | 0.78 |
| Previous flare number | 0.97 | 0.90-1.05 | 0.49 |
| MSU positive | 0.65 | 0.18-2.36 | 0.51 |
| ULT agents | | | |
| Allopurinol | Ref |  |  |
| Febuxostat | 2.42 | 0.93–6.27 | 0.07 |
| Benzbromarone | 10.92 | 1.33–106.73 | 0.03* |
| Combined medications | | | |
| Diuretics | 0.98 | 0.32-3.03 | 0.97 |
| Antihypertensive agents | 2.33 | 1.03–5.28 | 0.04* |
| Aspirin | 1.03 | 0.30-3.53 | 0.97 |
| Antiplatelet agent | 0.29 | 0.07-1.17 | 0.08 |
| Anticoagulant | 1.95 | 0.40-9.51 | 0.41 |
| Hypoglycemic agent | 0.65 | 0.23-1.83 | 0.42 |
| Statin | 1.90 | 0.70-5.17 | 0.21 |
| Initial serum uric acid | 0.90 | 0.72-1.12 | 0.33 |
| Adherence to ULT ≥ 80% | 12.31 | 2.46–61.67 | <0.01** |

**P*<0.05, ***P*<0.01

BMI, body mass index; BP, blood pressure; MSU, monosodium urate; ULT, urate-lowering therapy; XOI, xanthine oxidase inhibitor.
